# The development of communication in alarm contexts in wild chimpanzees

**DOI:** 10.1007/s00265-019-2716-6

**Published:** 2019-07-06

**Authors:** Guillaume Dezecache, Catherine Crockford, Klaus Zuberbühler

**Affiliations:** 1 Institut Jean Nicod, Département d’études cognitives, ENS, EHESS, CNRS, PSL Research University, Paris, France; 2 Budongo Conservation Field Station, Masindi, Uganda; 3 Department of Experimental Psychology, University College London, London, UK; 4 Department of Primatology, Max Planck Institute for Evolutionary Anthropology, Leipzig, Germany; 5 Institute of Biology, Université de Neuchâtel, Neuchâtel, Switzerland; 6 School of Psychology and Neuroscience, University of St Andrews, St Andrews, Scotland, UK

**Keywords:** Social learning, Social cognition, Alarm calling, Gaze, *Pan troglodytes*

## Abstract

**Abstract:**

Animals have evolved a range of communicative behaviours in the presence of danger. Although the mechanisms and functions of some of these behaviours have been relatively well researched, comparatively little is known about their ontogeny, including how animals learn to inform social partners about impending danger. In adult chimpanzees, behaviours in response to dangers involve several channels, particularly alarm calls and simultaneous gaze alternations with nearby recipients. Gaze alternations may allow inexperienced individuals to learn from more experienced ones by assessing their reactions to unfamiliar objects or events, but they may also provide the basis for more advanced social referencing. Here, we were interested in the development of these two common behaviours, alarm calling and gaze alternations, in wild chimpanzees (*Pan troglodytes schweinfurthii*) confronted with a threat. Using a cross-sectional design, we investigated those in 8 infant and 8 juveniles by experimentally exposing them to an unfamiliar but potentially dangerous object, a large, remotely controlled, moving spider model. For alarm calling, we found a positive relation with age, starting at around 28 months, although alarm calls were not consistently emitted until after 80 months. For gaze alternations, we found no age effect, with some of the youngest infants already showing the behaviour. Although its function remains unclear in infant and juvenile chimpanzees, gaze alternations emerge early in chimpanzee development. Alarm calling may require more advanced developmental stages, such as greater perceptual abilities, categorical capacities or more sophisticated social cognition, i.e. an understanding that danger is a collective experience that requires communication.

**Significance statement:**

Alarm calling and other anti-predatory behaviours have been the topic of much research but their ontogenies are still poorly described and understood. Recent studies on the behaviour of wild chimpanzees in threatening contexts have suggested sophisticated social cognitive abilities in adults. How do these behaviours develop in ontogeny? We addressed this question using a field experiment with 8 infants and 8 juveniles exposed to a novel and potentially threatening object in their natural habitat. We found that gaze alternations are present in some of the youngest individuals, potentially revealing early social awareness in chimpanzees. Age did not have an effect on the presence of gaze alternation. We also found that alarm calling was more common in older individuals, suggesting that call production and context of usage must be learnt. We discuss our results in light of developmental theories of social cognition and the role of social learning in the primate lineage.

**Electronic supplementary material:**

The online version of this article (10.1007/s00265-019-2716-6) contains supplementary material, which is available to authorized users.

## Introduction

As a general rule, developmental patterns of anti-predatory behaviours appear to depend on a species’ life history, with rapidly maturing species showing fully functional behaviours earlier than slowly maturing ones (Lea and Blumstein 2011). One well-studied anti-predator behaviour is ‘alarm calling’, a type of signal whose main function is to warn others of the presence of danger (Zuberbühler 2009; Klump and Shalter 2010). Alarm calls have been documented in many social animals where they lower the predation risk, not just for the actor, but often also for other group members (Caro 2005).

Whilst much research has examined its mechanisms and function, relatively less has been devoted to its development. Here, the majority of studies has focused on how individuals acquire appropriate responses to other group members’ alarm calls (reviewed in Hollen and Radford 2009; Lea and Blumstein 2011), with comparably less known about the acquisition of call production and context-dependent usage. One influential developmental model has emerged from field observations on free-ranging vervet monkeys (*Cercopithecus aethiops*). In this primate, individuals from a very early age produce acoustically distinct calls, albeit to a much broader set of events than adults. For instance, less than 12-month old infants can give aerial alarm calls to geese, pigeons or falling leaves, suggesting that any type of object from above initially triggers these calls. With experience, infants then learn to ignore events that do not pose a direct threat and will eventually only alarm call to a small range of dangerous predator species (Seyfarth and Cheney 1980). This developmental process is adaptive insofar as it enables individuals to develop habitat-specific anti-predator responses, whilst reducing the likelihood of costly false positives (Owings and Loughry 1985; Lea and Blumstein 2011). Although gradual changes in alarm call usage during infancy have also been reported in meerkats (*Suricata suricatta*) (Hollén and Manser 2006; Hollén et al. 2008), an alternative developmental model suggests that age-related differences in alarm calling behaviour reflect age differences in susceptibility to predation rather than individual experience (Hollén et al. 2008; Hollen and Radford 2009; Lea and Blumstein 2011).

Chimpanzees (*Pan troglodytes*) produce alarm calls to a range of disturbances, but previous studies have mainly focussed on a single class of threats—snakes. Chimpanzees emit three alarm call types to snakes: acoustically variable hoo alarm calls (different from hoos in non-predatory contexts; Crockford et al. 2018) (Schel et al. 2013), waa-barks and SOS screams (Goodall 1986; Zuberbühler 2000; Crockford and Boesch 2003; Schel et al. 2013; Crockford et al. 2018; Crockford 2019). The current literature suggests that these three call types are not strictly predator-specific, as demonstrated in various monkey species (e.g. Stephan and Zuberbühler 2016) but may reflect the degree of danger. Young chimpanzees show somewhat different vocal behaviours to dangers, by producing ‘hoo’ and ‘whimper’ calls (Plooij 1984; Plooij et al. 2015). Although the vocal behaviour of chimpanzees in threatening contexts has been the topic of much recent research (Crockford and Boesch 2003; Crockford et al. 2018; Crockford 2019), we are not aware of any systematic efforts to investigate the vocal behaviour of immature individuals. In adult chimpanzees, alarm call production is known to depend on a number of social parameters, such as presence of specific group members and their attentional and epistemic states (Crockford et al. 2012, 2017; Schel et al. 2013), suggesting that social competence (i.e. the history of social activities) is critical for the production of alarm calls. It is also undeniable that competence in alarm calling requires more basic cognitive competences, such as the ability to process and correctly identify predators and other dangers.

Another reason why chimpanzees are relevant for developmental studies is that they often complement alarm calling with conspicuous visual behaviour, gaze alternation, in ways that suggest awareness of their audience (e.g. Liebal et al. 2013; Schel et al. 2013). For example, adult chimpanzees can engage in gaze alternation between a danger and other group members (Schel et al. 2013; Crockford et al. 2017) often combined with alarm calling. Related to this, they have been observed to actively position themselves such that they can maintain visual contact to both the threat and other group members, a behaviour that has been termed ‘marking’ (Crockford et al. 2017), in line with a social referencing interpretation of gaze alternation (see also for positioning in other contexts; Liebal et al. 2004). It may function to obtain information from more experienced group members, as social referencing, allowing others to detect the threat (Schel et al. 2013; Crockford et al. 2017; Townsend et al. 2017), or as a means to establish joint attention, a necessary precondition for joint action, such as group-level predator mobbing or travelling (Gruber and Zuberbühler 2013). Gaze alternation has been documented both in captivity (Leavens and Hopkins 1998) and in the wild (Gruber and Zuberbühler 2013; Schel et al. 2013; Crockford et al. 2017). The alarm behaviour of chimpanzees may thus be considered to involve multimodal signals combinations, i.e., the coupling of two otherwise independent signals using distinct modalities (sensu Fröhlich and Hobaiter 2018).

In humans, gaze alternation has been studied extensively in the context of language acquisition, where it becomes frequent by 9 months. It is said to facilitate joint attention with adults and as such ease the acquisition of meaning (Carpenter et al. 1998). The behaviour may also facilitate social learning and provide a basis for more complex social interactions, such as gaze following, social referencing and joint attention (Evans and Tomasello 1986; Baldwin and Moses 1996; Striano and Rochat 2000; Slaughter and McConnell 2003; Tomasello et al. 2005).

In chimpanzees, gaze alternation has already been seen in 5-month-old infants (Bard et al. 2014) (but see Lucca et al. 2018). More generally, infant chimpanzees are socially responsive from 1 to 2 months of age (mutual gaze with mother), show sensitivity to gaze direction from 2 months, employ audience-checking (i.e. gazes at social partners) when producing communicative signals from 9 months and follow others’ gaze around 12 months of age (see Tomonaga et al. 2004 for a review; Fröhlich et al. 2018).

Although the presence of gaze alternations is undisputed in chimpanzees, their function is less clear, particularly in young individuals. Three competing hypotheses can be spelled out. One is that gaze alternations are not communicative devices but momentary shifts of attention driven directly by the environment. For example, an individual’s focus of attention may be momentarily disrupted by a novel event but then redirected back to the original focus. A second possibility (which also does not imply that their function is communication) is that gaze alternations allow one individual to check the availability of social partners. A last possibility is that the function of gaze alternations is communication, i.e. gaze alternations help direct the attention of audience members to an object worth of attention. In adults at least, and when interacting with dangers, gaze alternations appear to fulfil a communicative function (Schel et al. 2013; Crockford et al. 2017; Townsend et al. 2017), as they are more likely to occur in cooperative contexts, i.e. when audience members have most to gain (e.g. when those are not aware of a presence of a threat).

In this study, we were interested in the emergence of alarm calling and gaze alternation in young chimpanzees exposed to a threat. We studied the development of both behaviours in *N* = 16 young Eastern chimpanzees (*Pan troglodytes schweinfurthii*) (*N* = 8 infants, *N* = 8 juveniles) of the Sonso community of Budongo Forest, Uganda. To systematically examine the development in natural circumstances, we conducted controlled presentations of a model spider. This research contributes to our understanding of the development of social cognition, as alarm contexts represent situations where chimpanzees typically recruit their social partners, using visual and vocal signals (Crockford et al. 2012, 2017; Schel et al. 2013) and where call production is sensitive to audience affects due to the presence of kin or bond partners, especially if they are ignorant of the snake.

## Methods

### Study site

The study was carried out in the Budongo Forest Reserve, a moist, semi-deciduous, tropical forest in Western Uganda, covering 428 km^2^ at an altitude of 1100 m between 1° 35′ and 1° 55′ N and 31° 08′ and 31° 42′ E (Eggeling 1947). Data were collected on the Sonso chimpanzee community (Reynolds 2005) between January and April 2017. At the start of the study, the Sonso community was composed of 35 adults (> 15 years) (24 females), 7 subadults (> 11 years) (6 females), 14 juveniles (> 4 years) (10 females) and 11 infants (< 4 years) (2 females).

### Subjects

In chimpanzees, infancy is marked by physical dependence on the mother. Weaning begins at around 3–4 years when physical independence from the mother tends to increase. When weaned (at around 5 years), individuals are considered to be juveniles (Reynolds 2005; Laporte 2011). Although they may frequently travel with their mothers, juveniles gain physical independence and no longer ride their mothers dorsally, a behaviour seen in infants by 3 m.

In this study, we included all infants (individuals younger than 4 years) and juveniles (individuals older than 4 years and younger than 11 years) that were regularly seen. We were able to carry out experimental trials with 16 individuals (*N* = 8 infants; *N* = 8 juveniles; see Table 1 for details).

**Table 1:** List of subjects, sex, age (in months) and age class on the day of the experiment and responses: presence of an alarm call and gaze alternation

ID	Sex	Age (in months)	Age class	Alarm call (1 = yes)	Gaze alternation (1 = yes)
MZ	M	15	Infant	0	0
KV	M	25	Infant	0	1
OZ	M	28	Infant	1	1
KO	M	29	Infant	0	0
KF	M	36	Infant	0	0
HM	F	40	Infant	0	1
RY	M	41	Infant	1	1
KJ	M	44	Infant	0	1
HR	F	87	Juvenile	1	1
MB	M	97	Juvenile	0	0
KH	F	103	Juvenile	1	1
RF	F	115	Juvenile	1	0
KB	F	122	Juvenile	1	1
FA	F	124	Juvenile	1	1
KC	M	124	Juvenile	1	1
JS	M	129	Juvenile	1	1

### Experimental procedure

**Fig. 1:**
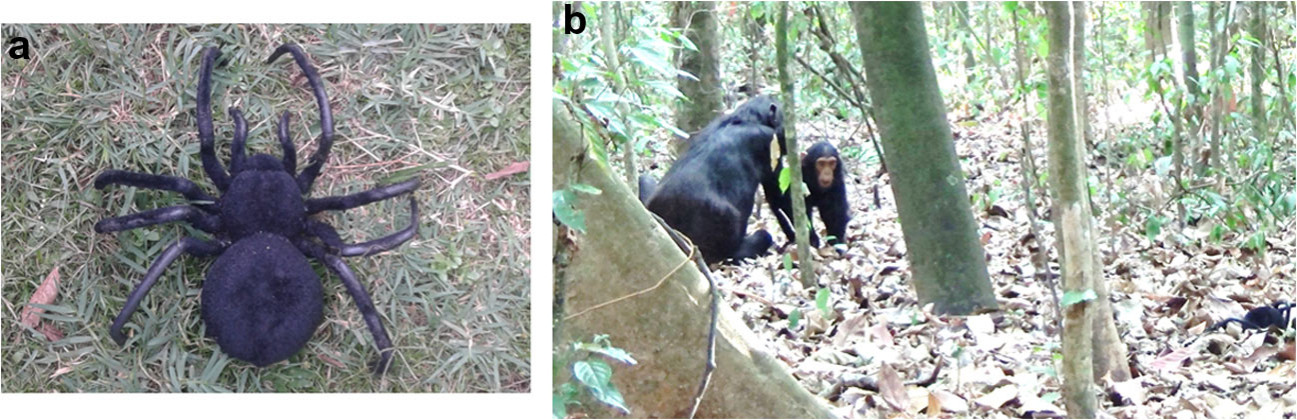
**a** The spider model used as unfamiliar, potentially hazardous experimental stimulus. **b** Usual setting of the experiment: individual OZ (infant) discovering a spider after following the gaze of his mother OK. He later approached the spider (far right), retreated suddenly and after re-establishing contact with the mother, emitted a soft alarm call

A mechanical object representing a spider (see Fig. 1a; 10.5 cm wide; 12 cm long) was presented to elicit alarm behaviour (see Fig. 1b). Motor-driven leg movements could be triggered by remote control. In a pilot experiment, we established that adult chimpanzees perceived the model as potentially hazardous. Presentation of the spider to four adult males elicited hoo alarm calls in all. The fact that there are no natural arachnoid species in Budongo Forest that even closely resembled the model, beyond its general spider-like physical appearance, was a deliberate design feature with two advantages. First, since subjects had never interacted with this particular device, they were unable to rely on previous experience to make predictions about its behaviour or potential dangerousness, regardless of age. Second, due to its unfamiliarity, the potential danger may have been interesting enough to be worth communicating about. The Sonso community occupies a home range that includes a dysfunctional former sawmill with a hamlet of houses that now serve as the base of the Budongo Conservation Research Station (Reynolds 2005). As a consequence, all individuals regularly encounter human artefacts, suggesting that our manipulation was within the natural range of experiences. To avoid habituation, we only performed one trial per subject. As subjects, we only accepted individuals that had never seen the model previously and were not within earshot of previous trials. We also tried to avoid that former subjects witnessed the model a second time as audience members. No human was ever seen interacting with the model by a chimpanzee.

In each trial, the spider was set at around 7 m from potential focal individuals by an experienced field assistant (Sam Adue), provided nearby chimpanzees were not attending. We could elicit detection (defined as a visual inspection of the spider) by remotely operating the model. The detecting infant or juvenile then became the subject. In all but one trial, infants or juveniles detected the spider before other audience members (i.e. individual OZ—see Table 1, detected the spider second, after following the gaze of his mother but nonetheless alarm called before the mother did). We videotaped the behaviour and commented on the focal individual until it moved away from and could no longer see the spider. We noted all other individuals present within 5, 10, 20 and 30 m of the focal animal and their relative position as left front, right front, right back and left back. Note that, in every trial, there was at least one individual (usually the mother or older sibling) within a radius of 30 m from the subject.

### Data coding and analysis

**Fig. 2:**
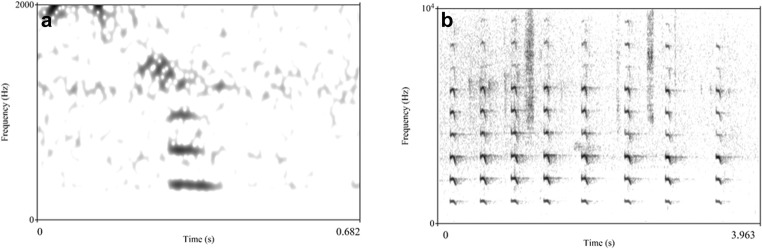
**a** Alarm call produced by individual JS (juvenile). Alarm call of young individuals resembles hoo alarm calls produced by adults: they are soft, tonal and low in frequency (Schel et al. 2013; Crockford 2019). In adults, repetition rates for hoo alarm calls are relatively slow (Crockford et al. 2018). **b** Series of distress calls produced by individual HR (juvenile). By contrast to hoo alarm calls, distress calls are higher in frequency and are produced at higher rate. Note difference in frequency scale on *y*-axis for **a** and **b**. The calls presented in **a** and **b** can be listened to at https://bit.ly/2J07yRs and https://bit.ly/2UIId0s, respectively

The presence or absence of the following behavioural elements were coded using BORIS (Friard and Gamba 2016) and Windows Media Player software, from the moment the spider was detected by the focal and up to 10 min after detection: (a) production of alarm call (yes/no), defined as calls resembling the hoo alarm call of adults, albeit sometimes softer. Young chimpanzees also produce whimpers (distress calls) when threatened (Plooij 1984), but those were not considered (see Fig. 2 for examples of alarm and distress calls in juveniles). We also considered (b) gaze alternation (yes/no), defined as alternating gaze between the spider, the direction of other group members and back to the spider, with no obvious breaks between gaze shifts (see Crockford et al. 2017).

All measures were coded by GD. Fourteen of the 16 trials were double-coded by an observer blind to the hypotheses. To distinguish alarm calls and whimpers, the double coder was trained on a set of calls before coding the data. We obtained 100% agreement on whether any of the following occurred: (a) alarm calling and (b) gaze alternation between spider and audience (κ = 1). Two of 16 trials (12.5%) could not be completely videotaped due to technical failure. However, both trials could be reconstructed in terms of the sequence of behaviour undertaken by the focal animal due to detailed records provided by the field assistant naïve to the hypotheses.

### Statistical analysis

We conducted logistic regression analyses. The presence or absence of alarm call and gaze alternation was used as dependent variables in two separate models. To check whether age influenced the outcome of the dependent variables, we used age in months as a predictor in our models. To control for unbalanced sex distributions (1 out of 8 infants is a female; 5 out of 8 juveniles are females), we also used sex as an independent variable in our models. We compared full models (including age and sex) to a corresponding null model (sex only), using likelihood ratio tests (LRT) (lrtest function of package lmtest, Version 0.9-36; Zeilis and Hothorn 2002). Age was entered in months. Note that since we only collected 1 trial per individual, ID was not considered as a random effect. We fitted all models in R (Version 3.5.1) (R Core Team 2018) with R Studio (Version 1.1.453) (RStudio Team 2016), using the glm function. Note that the following assumptions were met: the dependent variable in both models is dichotomous (presence or absence of alarm call or gaze alternation), there was linear relationships between the predictor (age) and logit of both outcomes and there were no influential values (i.e. data points with absolute standardized residuals above 3).

## Results

### Alarm calling

**Fig. 3:**
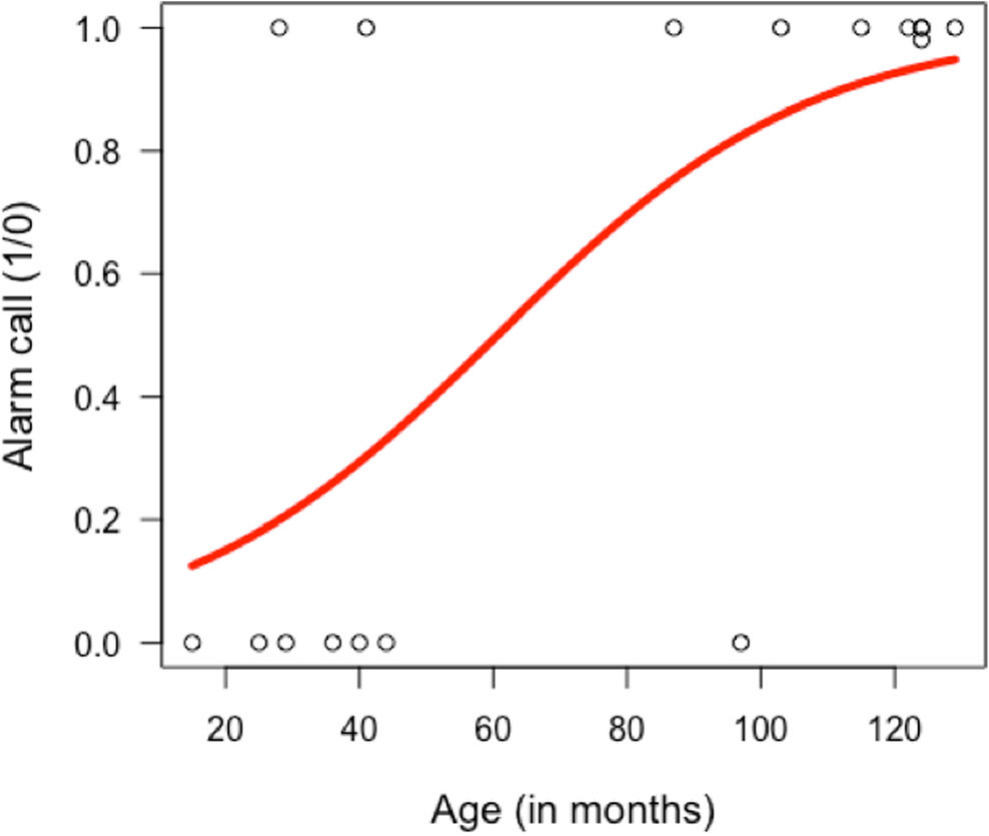
Alarm calling (*y*-axis; 1 = yes) as function of age in months (*x*-axis), with the predicted logistic curve (in red), using the R package popBio (version 2.4.4) and logi.hist.plot function (Stubben and Milligan 2007)

Age had a positive effect on the likelihood of producing an alarm call (alarm call emission 7 of 8 juveniles (87.5%) vs. 2 of 8 infants (25%); see Table 1; LRT *χ*^2^(1) = 5.24, *P* = 0.022) with individuals over 80 months more likely to emit alarm calls than younger individuals (Fig. 3).

### Gaze alternation

Age did not have an effect on the likelihood of producing at least one gaze alternation after detecting the stimulus (LRT *χ*^2^(1) = 0.25, *P* = 0.620) and was common in both infants and juveniles (5 of 8 infants (62.5%) and 6 of 8 juveniles (75%)).

## Discussion

Our study examined the ontogeny of anti-predator behaviour in wild chimpanzees using cross-sectional data. *N* = 16 individuals were exposed to a spider model that did not have a natural counterpart in their habitat but represented a generalized type of arachnoid. In a pilot experiment, the model trigged aversive reactions in adult males. As predicted, infants and juveniles reacted to the object in a manner that suggested they considered it to be a threat, by giving alarm and/or distress calls.

We were interested in the role of age in the development of alarm calling and gaze alternation. We found that older individuals were more likely to alarm call than others, suggesting that, in slowly maturing species, antipredator behaviour can be subject to learning. This may be in contrast to rapidly maturing species, such as certain species of sciurids, where infant anti-predator behaviour appears early and in an adult-like fashion (Lea and Blumstein 2011).

Why was alarm calling not observed more frequently in infants? One hypothesis is that infant chimpanzees can produce alarm calls from early on but that, in our experiment, the motivation to alarm call in younger individuals (who are usually in close proximity to their mothers) could have been hindered. When offspring are physically closer to the mother, a threat may trigger less arousal, or mothers may be more likely to see and react to the threat before the offspring.

Another hypothesis is that young infant chimpanzees do not regularly produce alarm calls because they rely on other vocal strategies, such as distress calling (Plooij 1984; Lingle et al. 2012), in order to devolve any antipredator actions to their mothers. Through development, as the physical proximity between mothers and their offspring increases and the choice of travel parties differs, juveniles are no longer able to rely on maternal assistance and will have to take a more active role when dealing with threats.

Also, the absence of alarm calling in younger individuals may reflect their inability to produce this particular vocal structure. Whilst hoo calls are present in the repertoire of very young chimpanzee infants (Plooij 1984), it remains unclear whether there exists context-specific hoo call variants as seen in adults (Crockford et al. 2018).

A last hypothesis is that infants lack the necessary knowledge to engage competently with unfamiliar objects, that is, a delay in what has been called ‘usage learning’ (Janik and Slater 2000). Here, the prediction is that young individuals first need to have experienced others calling in order to categorize the object as a threat. Although we employed a model that was equally novel to all individuals (including juveniles), it is undeniable that older individuals will have more experience allowing them to make generalizations based on shared physical features with other dangerous objects.

In environments that are degraded by human presence (Hockings et al. 2015), traits associated with wariness with novel objects may be beneficial, especially as many items associated with human activities (such as snares) represent a threat to chimpanzees in Budongo and elsewhere (Waller and Reynolds 2001; Quiatt et al. 2002). In Budongo, chimpanzees sometimes produce alarm calls upon encountering snares (Crockford et al. 2012). Flexibility and learning in the usage of alarm calls appear essential in chimpanzees, a species that live in a range of habitats, with specific predator fauna.

Our study also looked at the use of gaze alternations in infants and juveniles. For gaze alternation, we used a strict definition requiring that animals engaged in at least three sequential looks (to the spider, to the social partner, and back to the spider), whereas most previous studies typically required only two gazes (Bard et al. 2014). Nevertheless, we found that infants engaged regularly in gaze alternation (> 50% of trials), in contradiction to previous findings, which reported gaze alternation in late juveniles but not infants (Lucca et al. 2018). This highlights the importance of task type (danger vs. food), social partner (conspecific vs. human caretaker), living condition (wild vs. captivity) and behavioural definition of gaze alternation. Our results are more in line with another study that reports frequent gaze alternations by 5 months of age (Bard et al. 2014).

Which function does gaze alternations fulfil in infants and juveniles? In previous work (Schel et al. 2013; Crockford et al. 2017), it was proposed that gaze alternation in chimpanzees is a communicative behaviour, which helps audience members to acquire information about a potential danger in the environment. In our own study, the use of gaze alternation in younger individuals is equally consistent with a non-communicative function. It is indeed possible that gaze alternations help individuals check the availability of social partners for comfort seeking or for gaining information about the threat. By gaze alternating between the threat and audience members, younger individuals may gain information from the reactions of more experienced individuals about the nature of a threat, enabling social learning. This behaviour, in the human literature usually referred to as social referencing (Klinnert et al. 1983) is common and emerges in the first 2 years of life in humans (Walden and Ogan 1988), suggesting that it is evolutionarily old with counterparts in closely related non-human primates. Social referencing interpretations of gaze alternation, audience checking or audience monitoring has been invoked in chimpanzees (Ueno and Matsuzawa 2005), common marmosets (*Callithrix jacchus*; Kemp and Kaplan 2013), Barbary macaques (*Macaca sylvanus*; Roberts et al. 2008), domestic dogs (*Canis lupus familiaris*; Merola et al. 2012) and cats (*Felis catus*; Merola et al. 2015). In infant chimpanzees, social referencing has also been reported in interactions with human caregivers during exposure to a novel object (Russell et al. 1997). The social referencing hypothesis predicts that infants should look more towards older and mature individuals, including the mother, than younger and less knowledgeable individuals. A second prediction is that the subject’s reaction to the threat should be similar to the social referent’s reaction to the threat.

Another possibility is that gaze alternations in infants and juveniles are mere shifts in attention allocation caused by changes in the close environment (e.g. movements of another individual). Due to the density of the vegetation and the poor visibility, it is difficult to rule out this hypothesis with confidence. In two trials, however, visibility was good and there was no sign of movement or any other behavioural change in the audience that could have caused the gaze shift.

Regardless of its actual function in infants and juveniles, we found that gaze alternation is present from an early age in chimpanzees. Future studies should clarify its function.

## Limitations

Our research has provided new insights into the developmental unfolding of social behaviour in a threatening context in wild chimpanzees, whilst still under the care of their mothers. We found evidence for an effect of age on alarm calling but little influence of age on the emergence of gaze alternation, as this behaviour appears to emerge earlier in development (Bard et al. 2014). However, we see limitations to this research. Regarding statistical power, and although our sample size is comparable with other field experimental studies in great apes, replication in other sites appears important to secure the effect of age on the emergence of alarm calling.

Additionally, the sex ratios are not comparable between the infant and juvenile age classes (m/f infants 7/1 (9/2 possible); m/f juveniles 3/5 (4/10 possible)), to the effect that any sex-specific developmental differences are unlikely to be captured by our data. They yet matter, particularly in chimpanzees (Lonsdorf et al. 2018). Also, gaze alternation data from individuals younger than 15 months are missing to further evaluate the ontogenetic trajectory of this trait. With our methodology, however, it would probably be impossible to attract the attention of such young infants before that of the mother, suggesting that a new study design would be needed.

Another limitation is that we were not able to collect reliable data on the behaviour of the audience during the trials. In particular, it would have been very informative to investigate whether subjects looked more towards influential, experienced or familiar group members than other individuals. Mature individuals are more competent in assessing threat, suggesting that inexperienced individuals should preferentially seek visual contact with such individuals to gain valuable information. Also, as in other studies, the presence of kin and bond partners may have had a strong influence on the motivation to alarm call or to monitor other’s behaviour (Crockford et al. 2012, 2017; Schel et al. 2013). Additionally, coding others’ behaviour could clarify the function of gaze alternations, by assessing whether gaze alternation promotes corresponding shifts in the attentional state of audience members.

Similarly, it was also not possible to actively keep track of the behaviour of mothers upon detecting the spider. In primates, adult behaviour influences infants’ learning of predators, for example by sanctioning incorrect usage of alarm calls (Seyfarth and Cheney 1980), but we were not able to systematically investigate this here. On the contrary, in order to increase the probability that the focal animal discovered the object first (detectors are often the first to commit to the role of alarm caller in chimpanzees), we waited for audience members to be as far as possible from the focal (within a limit of 30 m) to start a trial.

## Conclusion

Despite its limitations, this study is a first experimental investigation of the ontogeny of alarm calling and gaze alternations in wild chimpanzees. It demonstrates that gaze alternations probably play a role in threat contexts in chimpanzees as young as 25 months old, although its function needs to be clarified. Our research also suggests that recognition of dangerous objects and associated alarm calling production may be subjected to social learning in chimpanzees. Finally, and beyond complementing new and important research on the development of communication in chimpanzees (Fröhlich et al. 2017, 2018; Lucca et al. 2018) and the reliance on social learning in the primate lineage (Maestripieri 2018; Whiten and van de Waal 2018), it represents a missing piece in an underdeveloped research area, that of the ontogeny of anti-predator behaviour.

## Electronic supplementary material

ESM 1 (DOCX 13 kb)

## Data Availability

The raw data is presented in Table 1.
