# Supplementary material for: The development of communication in alarm contexts in wild chimpanzees
Source: Behav Ecol Sociobiol. 2019 Jul 6;73(8):104. doi: 10.1007/s00265-019-2716-6 (PMC6612320; doi:10.1007/s00265-019-2716-6)
Supplement: Supplementary file 1 — (DOCX 13 kb) [file 265_2019_2716_MOESM1_ESM.docx]

**Electronic Supplementary Material (ESM) for “The development of communication in alarm contexts in wild chimpanzees” in *Behavioral Ecology and Sociobiology***

Guillaume Dezecache*, Catherine Crockford C. & Klaus Zuberbühler

*Corresponding author: [guillaume.dezecache@gmail.com](mailto:guillaume.dezecache@gmail.com)

Institut Jean Nicod, Département d’études cognitives, Pavillon Jardin, 29 rue d’Ulm, 75005 Paris;
+ 33(0)144322605; ORCID: 0000-0002-9366-6287

**Samples of chimpanzees’ behavioural reactions**

Please display the subtitles to be guided through the details.

Video 1 - OZ (infant) performing gaze alternation: <https://youtu.be/xnWcmQMmt3Y>

Video 2 - KB (juvenile) performing gaze alternation: <https://youtu.be/6bD9VMwhdJU>

Video 3 - JS (juvenile) alarm calling: <https://youtu.be/68WHtmJURdI>
